# Adverse Reactions in Relapsed/Refractory B-Cell Lymphoma Administered with Chimeric Antigen Receptor T Cell Alone or in Combination with Autologous Stem Cell Transplantation

**DOI:** 10.3390/cancers16091722

**Published:** 2024-04-28

**Authors:** Haolong Lin, Ting Deng, Lijun Jiang, Fankai Meng, Yang Cao, Yicheng Zhang, Renying Ge, Xiaojian Zhu

**Affiliations:** 1 Department of Hematology, Tongji Hospital, Tongji Medical College, Huazhong University of Science and Technology, Wuhan 430030, China; linhl@tjh.tjmu.edu.cn (H.L.); jianglj2024@163.com (L.J.); doctormeng@163.com (F.M.); caoyangemma@163.com (Y.C.); yczhang@tjh.tjmu.edu.cn (Y.Z.); 2 Immunotherapy Research Center for Hematologic Diseases of Hubei Province, Wuhan 430030, China; 3 Department of Hematology, Chongqing Fifth People’s Hospital, Chongqing 400062, China; dengting9965@163.com; 4 Department of Hematology, Xianning Central Hospital, The First Affiliated Hospital to Hubei University of Science and Technology, Xianning 437100, China

**Keywords:** B-cell lymphoma, CAR-T, ASCT, CRS, CRES

## Abstract

**Simple Summary:**

Chimeric antigen receptor T cell (CAR-T) therapy has demonstrated significant success in B-cell lymphoma. The combination with autologous stem cell transplantation (ASCT) further enhances the efficacy of CAR-T cell therapy, although its impact on adverse reactions remains inconclusive. This study examined 147 patients who received CAR-T alone and 145 patients who underwent ASCT combined with CAR-T for B-cell lymphoma. The combined group had a higher incidence of overall and grade 1–2 CRS, but the risk of grade 3–4 CRS and CRES was not elevated. A univariate logistic regression analysis revealed that females had a lower incidence of grade 3–4 CRS but a higher incidence of severe CRES. The involvement of the central nervous system also correlated with increased CRES occurrence. The findings suggest that combining ASCT with CAR-T does not heighten the risk of severe adverse effects post-infusion. However, further validation through multicenter, prospective clinical studies is necessary to establish CAR-T combined with ASCT as a superior treatment paradigm.

**Abstract:**

(1) Background: The combination of CAR-T with ASCT has been observed to enhance the efficacy of CAR-T cell therapy. However, the impact of this combination on adverse reactions is still uncertain. (2) Methods: Between January 2019 and February 2023, 292 patients diagnosed with r/r B-cell lymphoma received either CAR-T therapy alone or in combination with ASCT at our institution. We evaluated the incidence of CRS and CRES and utilized a logistic regression model to identify factors contributing to severe CRS (grade 3–4) and CRES (grade 3–4). (3) Results: The overall incidence of CRS and CRES was 78.9% and 8.2% in 147 patients receiving CAR-T alone, and 95.9% and 15.2% in 145 patients receiving CAR-T combined with ASCT, respectively. The incidence of overall CRS (*p* < 0.0001) and mild CRS (grade 1–2) (*p* = 0.021) was elevated in the ASCT combined with CAR-T group. No significant difference was observed in severe CRS and CRES between the groups. Among the 26 cases of lymphoma involving the central nervous system (CNS), 96.2% (25/26) developed CRS (15.4% grade 3–4), and 34.6% (9/26) manifested CRES (7.7% grade 3–4). Female patients had a lower incidence of severe CRS but a higher incidence of severe CRES. Lymphomas with CNS involvement demonstrated a higher risk of CRES compared to those without central involvement. (4) Conclusions: The combination of ASCT with CAR-T demonstrated a preferable option in r/r B-cell lymphoma without an increased incidence of severe CRS and CRES.

## 1. Introduction

B-cell lymphoma comprises a group of malignant tumors originating from lymph nodes and lymphoid tissues, characterized by high heterogeneity and diverse manifestations. While the combination of rituximab with standard chemotherapy has yielded positive outcomes, conventional treatments remain ineffective for individuals with highly aggressive and refractory/recurrent (r/r) B-cell lymphoma. Notably, recurrent drug resistance accounts for mortality in 20% of patients [1]. Autologous stem cell transplantation (ASCT) stands as a crucial modality for consolidation or intensive therapy following remission. Nevertheless, a subset of patients may experience relapse even after undergoing ASCT [2,3,4]. However, the prognosis for patients with relapsed/refractory (r/r) disease or those deemed unsuitable for ASCT remains notably bleak.

In recent years, the advent of chimeric antigen receptor T cell (CAR-T) therapy has heralded a groundbreaking development, offering newfound optimism for individuals grappling with r/r B-cell lymphoma. Documented results indicate that CAR-T therapy has shown a notably elevated overall response rate (ORR) following the ineffectiveness of conventional therapeutic approaches [5,6,7,8]. Several clinical trials have showed that CAR-T cell therapy can advance to patients who are resistant to frontline standard treatment or relapse within 12 months [9,10,11,12]. Additionally, adverse reactions in CAR-T cell therapy, notably cytokine release syndrome (CRS) and CAR-T cell-associated encephalopathy syndrome (CRES) cannot be ignored. These side effects may pose life-threatening risks for some patients and hinder the progress and application of CAR-T cell therapy [13]. Therefore, further exploration of a more effective and safer approach for r/r B-cell lymphoma is imperative.

Our center offers two treatment protocols for r/r B-cell lymphoma: a combination of ASCT and CAR-T, and CAR-T cell therapy alone. We found that CAR-T therapy following ASCT could achieve higher ORR, complete remission rate (CRR), progression-free survival (PFS), and overall survival (OS) [14,15,16,17]. Furthermore, two studies have substantiated the superiority of this novel combination therapy over CAR-T monotherapy in r/r B-cell lymphoma in other institutions [18,19]. Theoretically, myeloablative pretreatment before ASCT can alleviate tumor burden and diminish the immunosuppressive tumor microenvironment. The infusion of CAR-T cells during hematopoietic reconstitution further eliminates residual lesions and decreases suppressed immune regulatory factors such as monocytes and macrophages. As monocytes and macrophages are primary sources of IL-6, IL-1, and nitric oxide (NO), contributing to CRS, the integration of ASCT with CAR-T may potentially reduce the incidence of CRS and CRES following CAR-T cell infusion [20,21]. Nevertheless, there is currently no comprehensive description and comparison of CRS and CRES between the two treatment protocols.

In this study, a retrospective analysis was conducted on the incidence of CRS and CRES in 292 patients with r/r B-cell lymphoma who underwent either ASCT combined with CAR-T or CAR-T cell therapy alone. The findings from this analysis aim to provide valuable insights for these patients in making informed decisions regarding their future treatment choices.

## 2. Materials and Methods

### 2.1. Enrollment

A retrospective analysis encompassed 292 patients diagnosed with r/r B-cell lymphoma who underwent CAR-T cell therapy alone or ASCT combined with CAR-T at the Department of Hematology, Tongji Hospital, Tongji Medical College, Huazhong University of Science and Technology, from January 2019 to February 2023 (details are available in Supplement Table S1). All patients met the following inclusion criteria: (1) all enrolled cases underwent biopsy histopathology and immunohistochemistry testing, and met the diagnostic criteria outlined in the 2016 revision of WHO classification of B-cell lymphoma classification [22]; (2) relapsed/refractory to their prior treatments (therapy lines ≥ 2); (3) tumor cells expressing CAR-T-targeted antigens confirmed by flow cytometry or immunohistochemistry; (4) good Eastern Cooperative Oncology Group performance status (≤2); (5) a life expectancy of 12 weeks or longer. Patients with other malignancies or severe organ dysfunction prior to CAR-T infusion were excluded.

The CAR-T cells were provided by Wuhan Bio-Raid Biotechnology Co., Ltd. (Wuhan, China), consisting of a single-chain variable fragment, two co-stimulatory domains (CD28 and 4-1BB), and the intracellular CD3ζ activation domain. All CAR-T cell products passed quality control.

The study received approval from the Medical Ethics Committee of Tongji Hospital, Tongji Medical College, Huazhong University of Science and Technology, in adherence to the principles of the Helsinki Declaration. Clinical characteristics and laboratory data, including age, gender, tumor classification, targets of CAR-T therapy, and CRS as well as CRES after CAR-T cell transfusion, were extracted from the electronic medical record system.

### 2.2. Study Design

CRS and CRES were managed based on a grading system according to the expert consensus and guidelines [23]. There were 147 patients receiving CAR-T cell therapy alone and 145 patients received combined therapy of ASCT and CAR-T. The incidences of CRS and CRES following CAR-T cells reinfusion were meticulously observed in both cohorts.

### 2.3. Statistical Analysis

GraphPad Prism 8.3.0 and SPSS 26.0 statistical software were utilized for data processing, analysis, and visualization. Initially, normality testing was conducted for the measurement data. Data following a normal distribution (*p* > 0.05) were presented as (x ± s), while non-normally distributed data (*p* < 0.05) were represented as the median (minimum–maximum). Non-parametric tests were applied based on group distinctions. Enumeration data were expressed as absolute count and percentage [*n* (%)], and analyzed with the chi-square test. The univariate logistic regression model was employed to scrutinize factors influencing the occurrence of severe CRS and CRES. A significance level of *p* < 0.05 was considered indicative of statistical significance.

## 3. Results

### 3.1. Clinical Features

In the cohort of 292 cases with r/r B-cell lymphoma, the median subject age was 48 years (range 14–75), and the male-to-female ratio stood at 175:117. The distribution of lymphoma subtypes included diffuse large B-cell lymphoma (DLBCL, *n* = 256), Hodgkin’s lymphoma (*n* = 9), follicular lymphoma (*n* = 5), mantle cell lymphoma (*n* = 6), Burkitt lymphoma (*n* = 11), high-grade B-cell lymphoma (*n* = 4), and B-cell lymphoblastoma (*n* = 1). Among these cases, 26 patients had central nervous system lymphoma. Specifically, 147 patients underwent CAR-T therapy alone, while 145 patients received a combination of ASCT and CAR-T. Regarding CAR-T specifics, CD19 CAR-T cells were infused in 63 cases, CD22 CAR-T cells in 3 cases, CD30 CAR-T cells in 9 cases, combined infusion of CD19/22 CAR-T cells in 164 cases, CD19/20 CAR-T cells in 34 cases, CD20/22 CAR-T cells in 4 cases, and CD19/20/22 CAR-T cells in 15 cases. The median age in the combined group was notably lower than that in the CAR-T alone group (*p* < 0.01). However, there were no significant differences observed in variables such as age (≥60 years or not), gender, pathological type of lymphoma, and target of CAR-T cells between the two groups (*p* > 0.05) (Table 1).

In the CAR-T monotherapy group, the median duration of CRS was 5 days (range 1–30 days), with an average duration of 6.5 days. The median time for the onset of the first CRS of any grade following CAR-T cells infusion was 2 days (range 1–20 days), with an average of 4 days. The median duration of CRES was 2 days (range 2–6 days), with an average duration of 2.9 days. The median time for the onset of the first nervous system adverse event (AE) of any grade was 7 days (range 2–12 days) following CAR-T cell infusion and 4 days (range 1–8 days) post-CRS, respectively.

In the combined group of ASCT and CAR-T cells, the median duration of CRS was 7 days (range 2–26 days), with an average duration of 8 days. The median time for the onset of the first CRS of any grade following CAR-T cell infusion was 2 days (range 1–11 days), with an average of 2.5 days. Concerning CRES, the median duration was 5 days (range 1–20 days), with an average duration of 5.6 days. The median time for the onset of the first nervous system AE of any grade was 6 days (range 1–17 days) following CAR-T cell infusion and 4 days (range 1–17 days) post-CRS, respectively.

### 3.2. Incidence of CRS and CRES

**Figure 1:**
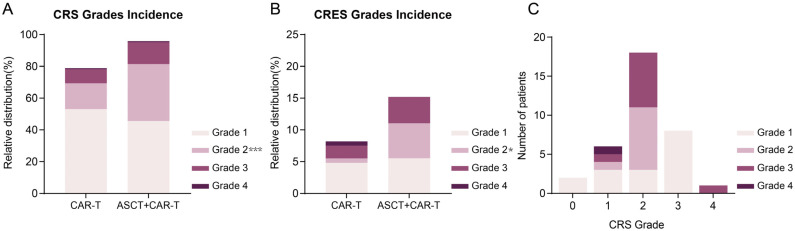
The incidence of CRS and CRES of each grade in two groups. (**A**). The incidence of CRS of each grade in CAR-T monotherapy group and combined group of ASCT and CAR-T (*, *p* < 0.05; ***, *p* < 0.001). (**B**). The incidence of CRES of each grade in CAR-T monotherapy group and combined group of ASCT and CAR-T. (**C**). The number of patients with CRES in each grade with CRS.

The incidence of CRS and CRES in the CAR-T monotherapy group was 78.9% and 8.2%, respectively, and rose to 95.9% and 15.2% in the group undergoing ASCT combined with CAR-T. In the CAR-T monotherapy group and the combined treatment group, the incidence of mild CRS (grade 1–2) was 69.4% and 81.4%, while that of severe CRS (grade 3–4) was observed in 9.5% and 14.5% cases, respectively. Regarding CRES, mild cases occurred in 5.5% in the monotherapy group and 11.1% in combined treatment group, while severe cases were reported in 2.7% and 4.1% of the two groups, respectively. The incidence of overall CRS (*p* < 0.0001) and mild CRS (*p* < 0.05) was higher in the combined treatment group than in the CAR-T alone group. However, no significant differences were observed in the incidence of severe CRS or CRES between the two groups (Table 2 and Figure 1A,B).

A total of 26 lymphoma patients exhibited central nervous system (CNS) involvement (3 in the CAR-T monotherapy group and 23 in the combined therapy group). Among these cases, 25 (96.2%) experienced CRS, with 15.4% classified as grade 3–4. Additionally, 9 cases (34.6%) manifested CRES, and 2 cases (7.7%) were characterized by grade 3–4.

### 3.3. Analysis of Factors Affecting the Occurrence of CRS and CRES

A univariate logistic regression analysis indicated that female patients exhibited a lower incidence of severe CRS but a higher incidence of severe CRES. However, age, lymphoma pathology, and target of CAR-T showed no correlation with the incidence of severe CRS and CRES (Table 3). Notably, lymphomas involving the CNS were more prone to developing CRES compared to those without central involvement.

## 4. Discussion

Great changes have taken place in the treatment of r/r B-cell lymphoma, and CAR-T cell therapy is the most promising one [24]. CAR-T cell therapy involves the construction of a specific chimeric antigen receptor (CAR) and then inducing T cells to express the CAR with genetic engineering technology. This process enables T cells to selectively identify and eliminate tumor cells. With deeper insights into lymphoma mechanisms and the tumor microenvironment [25,26], CAR-T cell therapy has demonstrated substantial success. In recent years, numerous medical centers have implemented ASCT followed by CAR-T cell therapy in the treatment of r/r B-cell lymphoma, yielding promising outcomes [14,15,19,27]. Compared to CAR-T monotherapy, the combined therapy of CAR-T and ASCT offers notable advantages, significantly enhancing the clinical prognosis for patients who show insensitivity to salvage chemotherapy [14]. Nevertheless, challenges persist in this treatment paradigm, including recurrence and treatment-associated adverse reactions [28].

CRS stands as the predominant adverse reaction in CAR-T cell therapy. Previous clinical investigations into CAR-T treatment revealed that the majority of subjects experienced CRS to varying grades. Remarkably, over 80% of patients exhibited grade 1–2 CRS without necessitating clinical intervention. A mere 10% to 20% of patients encountered grade 3–4 CRS. Only 10% to 20% of patients experienced grade 3–4 CRS, requiring targeted treatment [29].

In our study, the overall incidence of CRS in the CAR-T monotherapy group and combined group was 78.9% and 95.9%, with 69.4% and 81.4% of grade 1–2 CRS and 9.5% and 14.5% of grade 3–4 CRS, respectively. These findings are in general agreement with previously reported data [14]. Existing studies suggest that the severity of CRS is correlated with factors such as the number of infused CAR-T cells, the peak number of CAR-T cells in the blood [5], serum cytokine (including IFN-γ, TNF-α, IL-6, etc.) and C-reactive protein levels [30,31].

The median onset time for the first nervous system AE of any grade after CAR-T cell infusion was 7 days in the CAR-T monotherapy group and 6 days in the combined therapy group, respectively. This suggests that neurotoxicity may manifest concurrently with CRS or independently [32,33]. In this study, the factors related to the severity of CRS and CRES were further explored. It was revealed that the incidence of grade 3–4 CRS was lower but the incidence of severe CRES was higher in females. Nevertheless, no correlations were identified between age, lymphoma pathological type, targets of CAR-T, and the incidence of severe CRS or CRES. However, the potential impact of a small sample size for certain lymphoma pathological types and specific CAR-T targets cannot be disregarded (Supplemental Figure S1). No significant correlation was observed between neurotoxicity and the occurrence of CRS [OR 0.4 (0.09, 1.74) *p* = 0.22] or its severity [OR 1.23 (0.15, 10.04) *p* = 0.84]. Among the 10 patients with grade 3–4 CRES, only one patient had grade 3–4 CRS, which was inconsistent with previous reports [34]. Their average age was 49.6 years, similar to the overall cohort’s average age, but with females accounting for 80%, significantly higher than the overall female proportion (40.1%). Nevertheless, among all 34 patients experiencing neurotoxicity, with an average age of 49.7 years and females accounting for 38.2%, 32 exhibited at least grade 1 CRS accompanied by fever (≥38 °C) before the onset of neurological symptoms (Figure 1C).

Prior studies have affirmed the efficacy and manageable side effects of combining ASCT with CAR-T in the treatment of CNS lymphoma [35]. This could be attributed to the diminished tumor burden post-transplantation and the amelioration of the tumor immunosuppressive microenvironment within the CNS. Among 26 lymphoma patients with central involvement (23 received CAR-T therapy combined with ASCT) in our study, 9 (34.6%) developed CRES, including 2 cases with grade 3–4 CRES (7.7%). Notably, central involvement in lymphomas increased the risk of developing CRES, which may be explained by the disruption of the blood–brain barrier and heightened vascular permeability [33].

The majority of patients experiencing CRS and CRES typically present with mild to moderate symptoms that often resolve without drug interventions, such as dexamethasone or tocilizumab. Instances of life-threatening CRS and CRES are rare. There was no significant difference in the incidence of grade 3–4 CRS and CRES between combined therapy group and CAR-T monotherapy group in our study. Previous reports have suggested that CAR-T combined with ASCT provides superior efficacy and a lower recurrence rate in r/r B-cell lymphoma. This phenomenon may be ascribed to the myeloablative environment induced by ASCT, which stimulates specific CD8+ T cell expansion after CAR-T reinfusion and triggers an immune response, thereby enhancing the anti-tumor effect [36].

## 5. Conclusions

In summary, the combination of CAR-T cell therapy with ASCT does not increase the occurrence of grade 3–4 CRS and CRES in r/r B-cell lymphoma patients, suggesting it as a preferable option for treatment. However, further comprehensive research is warranted to investigate strategies for preventing and managing adverse reactions linked to CAR-T therapy, thereby improving its safety profile.

## Figures and Tables

**Table 1:** Patients baseline and clinical characteristics.

Characteristic	CAR-T (N = 147)	ASCT + CAR-T (N = 145)	*p* Value
	number of patients (percent)		
Age, years			
Median	49.8 ± 12.4	44.8 ± 12.1	0.01
<60	115	124	0.133
≥60	32	21	
Sex			0.981
Female	59 (40.1)	58 (40.0)	
Male	88 (59.9)	87 (60.0)	
Pathological type			0.151
DLBCL	128 (87.1)	128 (88.2)	
HL	7 (4.8)	2 (1.4)	
FL	3 (2.0)	2 (1.4)	
MCL	3 (2.0)	3 (2.1)	
Burkitt Lymphoma	2 (1.4)	9 (6.2)	
HGBL	3 (2.0)	1 (0.7)	
B-LBL	1 (0.7)	0 (0)	
CNSL			<0.0001
yes	3 (2.0)	23 (15.9)	
no	144 (98.0)	122 (84.1)	
Targets			0.294
CD19	32 (21.7)	31 (21.4)	
CD22	0 (0)	3 (2.1)	
CD30	7 (4.8)	2 (1.4)	
CD19/22	78 (53.1)	86 (59.3)	
CD19/20	20 (13.6)	14 (9.6)	
CD20/22	2 (1.4)	2 (1.4)	
CD19/20/22	8 (5.4)	7 (4.8)	

DLBCL: diffuse large B-cell lymphoma; HL: Hodgkin lymphoma; FL: follicular lymphoma; MCL: mantle cell lymphoma; MZL: marginal zone lymphoma; HGBL: high-grade B-cell lymphoma; B-LBL: B-cell lymphoblastic lymphoma; CNSL: central nervous system lymphoma.

**Table 2:** Cytokine release syndrome and CAR-T cells-related encephalopathy syndrome with treatments.

Item	CAR-T (N = 147)	ASCT + CAR-T (N = 145)
	number of patients (percent)	
CRS		
Grade 1	78 (53.1)	66 (45.5)
Grade 2	24 (16.3)	52 (35.9)
Grade 3	13 (8.8)	20 (13.8)
Grade 4	1 (0.7)	1 (0.7)
CRES		
Grade 1	7 (4.8)	8 (5.52)
Grade 2	1 (0.7)	8 (5.52)
Grade 3	3 (2.0)	6 (4.14)
Grade 4	1 (0.7)	0 (0)

**Table 3:** Univariate logistic regression analysis of the incidence of grade 3–4 CRS and CRES.

Item	CRS		CRES	
	OR (95%CI)	*p*	OR (95%CI)	*p*
**Age, years**	0.962 (0.922–1.003)	0.067	1.012 (0.941–1.089)	0.743
**Age** (<60 vs. ≥60)	1.529 (0.408–5.732)	0.529	0.845 (0.097–7.322)	0.879
**Age range**				
0–30	1		1	
31–40	0.027 (0.000–5.377)	0.181	-	0.995
41–50	0.049 (0.001–2.629)	0.138	-	0.999
51–60	0.085 (0.005–1.439)	0.088	-	0.996
≥60	0.337 (0.062–1.844)	0.210	-	0.996
**Sex** (Male vs. Female)	0.389 (0.162–0.935)	0.035	8.060 (1.513–42.95)	0.014
**Treatment type** (CAR-T vs. ASCT + CAR-T)	1.327 (0.595–2.962)	0.490	1.737 (0.434–6.953)	0.435
**Pathological type**				
DLBCL	0.717 (0.139–3.698)	0.691	-	0.999
HL	-	0.999	-	1
FL	-	0.999	-	0.999
MCL	2.174 (0.201–23.548)	0.523	-	1
Burkitt Lymphoma	1		1	
HGBL	1.812 (0.107–31.044)	0.679	-	1
B-LBL	-	1	-	1
**CNSL** (No vs. Yes)	0.902 (0.150–5.440)	0.910	5.151 (0.405–65.592)	0.207
**Targets**				
CD19	1.524 (0.586–3.962)	0.388	1.760 (0.431–7.194)	0.431
CD22	6.782 (0.511–89.934)	0.147	-	0.999
CD30	-	0.999	-	1
CD19/22	1		1	
CD19/20	2.644 (0.979–7.140)	0.055	-	0.999
CD20/22	-	0.999	-	0.999
CD19/20/22	-	0.999	-	0.999

DLBCL: diffuse large B-cell lymphoma; HL: Hodgkin lymphoma; FL: follicular lymphoma; MCL: mantle cell lymphoma; MZL: marginal zone lymphoma; HGBL: high-grade B-cell lymphoma; B-LBL: B-cell lymphoblastic lymphoma; CNSL: central nervous system lymphoma.

## Data Availability

The additional data collected in this study are available from the corresponding authors on reasonable request.

## Supplementary Materials

The following supporting information can be downloaded at: https://www.mdpi.com/article/10.3390/cancers16091722/s1, Table S1: Detailed Information of Enrolled Patients. Figure S1: The distribution of CRS and CRES grades among patients with different pathological types of B-cell lymphoma under the two treatment protocols.
