# Supplementary material for: Adverse Reactions in Relapsed/Refractory B-Cell Lymphoma Administered with Chimeric Antigen Receptor T Cell Alone or in Combination with Autologous Stem Cell Transplantation
Source: Cancers (Basel). 2024 Apr 28;16(9):1722. doi: 10.3390/cancers16091722 (PMC11083715; doi:10.3390/cancers16091722)
Supplement: Supplementary file 1 [file cancers-16-01722-s001.zip › Supplemental Figure S1. The distribution of CRS and CRES grades among patients with different pathological types of B-cell lymphoma under the two treatment protocol.pdf]

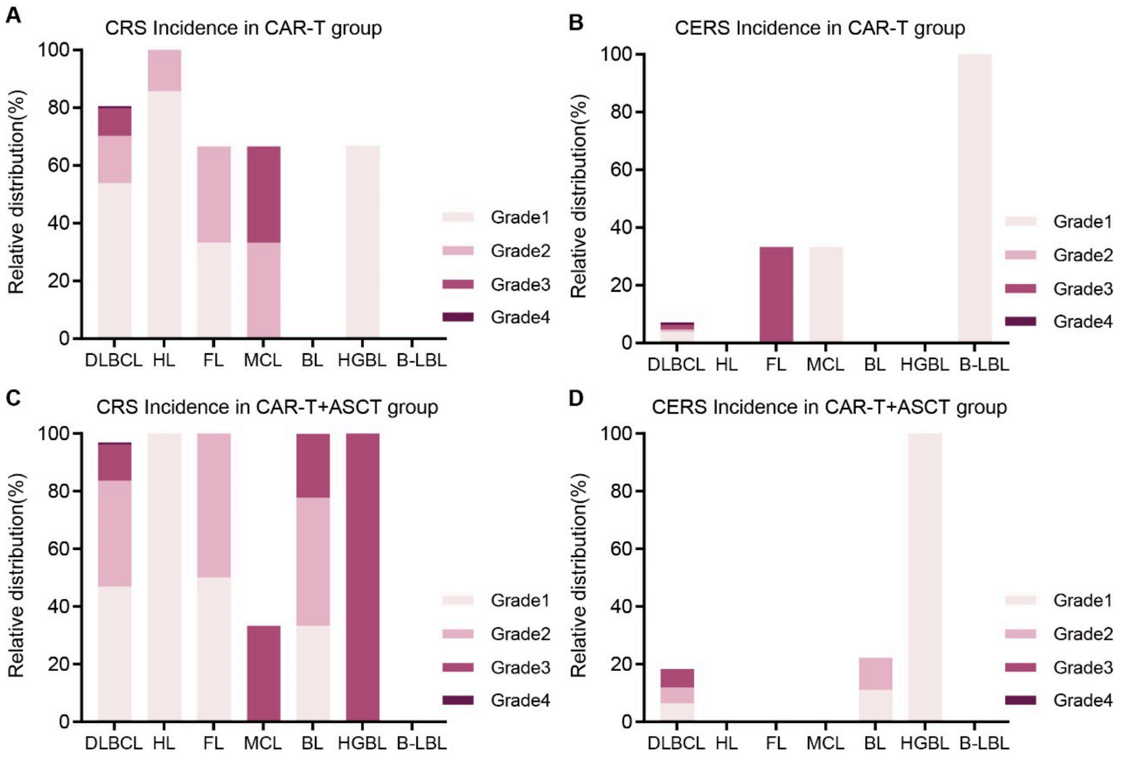

Supplemental Figure S1. The distribution of CRS and CRES grades among patients with different pathological types of B-cell lymphoma under the two treatment protocols.

A. The incidence of CRS grade among different pathological types of B-cell lymphoma in CAR-T group.

B. The incidence of CRES grade among different pathological types of B-cell lymphoma in CAR-T group.

C. The incidence of CRS grad among different pathological types of B-cell lymphoma in CAR-T combined with ASCT group.

D. The incidence of CRES grade among different pathological types of B-cell lymphoma in CAR-T combined with ASCT group.
